# No evidence for a relationship between MHC heterozygosity and life history strategy in a sample of North American undergraduates

**DOI:** 10.1038/s41598-020-67406-7

**Published:** 2020-06-23

**Authors:** Damian R. Murray, James B. Moran, Marjorie L. Prokosch, Nicholas Kerry

**Affiliations:** Department of Psychology, Tulane University, 2007 Percival Stern Hall, New Orleans, LA 70118 USA

**Keywords:** Behavioural genetics, Human behaviour

## Abstract

Although allelic diversity at the major histocompatibility complex (MHC) has implications for adaptive immunity, mate choice, and social signalling, how diversity at the MHC influences the calibration of life history strategies remains largely uninvestigated. The current study investigated whether greater MHC heterozygosity was associated with markers of slower life history strategies in a sample of 789 North American undergraduates. Contrary to preregistered predictions and to previously published findings, MHC heterozygosity was not related to any of the psychological life history-relevant variables measured (including short- vs. long-term sexual strategy, temporal discounting, the Arizona life history battery, past and current health, disgust sensitivity, and Big Five personality traits). Further, no meaningful effects emerged when analysing women and men separately. Possible reasons for why the current results are inconsistent with previous work are discussed.

## Introduction

The major histocompatibility complex (MHC)—a set of highly polymorphic genes within vertebrates—has received significant research attention in the behavioural sciences in recent years (e.g.,^1–8^). While the MHC is crucial for adaptive immunity and self versus non-self antigen recognition in vertebrates (e.g.,^9^), it has also been implicated in mate choice, kin recognition, and other social signalling contexts in both human and non-human animals (see^5,10^). Much of this work suggests a potential “heterozygote advantage” pertaining to the MHC, whereby greater allelic diversity at the MHC confers greater adaptive immunity and thus lower vulnerability to multiple pathogen threats^11–13^.

More recent research has found evidence for a link between greater MHC heterozygosity and slower life history strategies in humans^14^. This work is theoretically underpinned by the heterozygote advantage framework above, which logically implies that the better adaptive immunity conferred by MHC heterozygosity confers lower extrinsic mortality risk which, in turn, leads to the adoption of slower life history strategies. However, this research is limited in several ways, including the use of a small, women-only sample and measuring only sexually-related indicators of life history strategy. Here, we more fully investigate the potential link between MHC diversity and life history strategy using multiple indicators of life history strategy in a larger sample.

### The major histocompatibility complex

The MHC (or the Human Leukocyte Antigen system in humans) plays a critical role in pathogen recognition and immune response, largely through encoding cell surface molecules involved in presenting antigenic peptides to immune cells (see^15^ for a review of MHC function). The MHC is the most highly polymorphic region of the genome, containing hundreds of allelic variants for each MHC gene. This high polymorphism was most likely selected for (and is conserved) due to the antigen recognition-based benefits of heterozygosity (MHC alleles are expressed codominantly, so heterozygosity increases the variety of immune-related cell surface molecules; see^11,16^). Several lines of human and non-human research provide support for the ‘heterozygote advantage’ perspective of MHC diversity (e.g.,^12,13,17–21^). However, some models suggest that the increased T-cell repertoire depletion and the higher risk of autoimmune disorders associated with greater MHC diversity creates selection for an *optimum* number of different MHC molecules, rather than unconditional positive selection for heterozygosity (e.g.,^22,23^). Indeed, some studies have found evidence for an intermediate level of heterozygosity being optimal (e.g.,^24–27^). Other investigations have found no evidence for a relationship between MHC diversity and host resistance (e.g.,^28–30^), and some alternatively find evidence for strong selective pressure on specific alleles due to specific ecologically-dependent pathogens (e.g.,^31^). Thus, evidence for the heterozygote advantage across contexts remains equivocal.

Heterozygosity also appears to have implications for mate choice, such that individuals may prefer MHC-dissimilar mates in order to increase offspring heterozygosity. Dissimilarity preferences have been found across a range of non-human animals^32–36^, and some work suggests MHC-dissimilarity is associated with higher fecundity (e.g.,^1,2^). In humans, some studies suggest that members of MHC-similar romantic couples are less sexually responsive to their partners and report higher levels of extra-pair attraction^37,38^. Other studies have found that individuals prefer the odours and faces of MHC-dissimilar others (^3,39–41^, although cf.^42^). Some population-level studies find evidence for MHC-disassortative mating e.g.^43–45^ whereas others do not^46,47^.

MHC heterozygosity may also be associated with physical attractiveness. Results from several studies suggest that both the faces and body odours of more heterozygous opposite-sex targets are rated as more attractive (e.g.,^3,39,48^). However, these results are more equivocal for women’s versus men’s heterozygosity (see also^49^). Thus, while it appears that MHC influences mate choice in humans, the exact parameters of this influence remain unclear (see^50^ for review).

### MHC and life history strategies

Life history theory is a framework designed to capture how organisms strategically allocate finite time and energy resources in ways that are most likely to optimize fitness within the constraints of their local environment. “Faster” strategies (favouring earlier reproductive timing and current versus future payoffs) are typically more adaptive in harsher and more uncertain environments, given that delaying payoffs when survival is uncertain has diminishing returns^51–57^. Consistent with these Life History Theory predictions, studies have found that individuals who grow up in environments higher in extrinsic mortality threats tend to adopt faster life history strategies (see^56,58–64^).

One such extrinsic mortality threat influencing life history calibration is the threat of disease. People in ecologies characterized by higher infectious disease prevalence display less nurturant and investing parenting, consistent with the characteristics of a faster life history strategy^65^, and similarly pursue faster mating and reproductive strategies^66^. Similarly, small scale societies inhabiting regions of higher pathogen prevalence are significantly more likely to favour polygynous—rather than monogamous—marriage systems^67^.

Notably, however, an individual’s risk of mortality from infectious disease is not determined solely by the magnitude of pathogenic threats in the environment (i.e. *external* to the individual); it is also determined by the individual’s immunological profile and other *internal* factors. All else equal, individuals with a poorer ability to defend themselves from pathogens are more vulnerable to infectious disease, and therefore more susceptible to these extrinsic, unpredictable threats. Pursuing a faster life history strategy in these circumstances may historically have served to increase individuals’ chances of reproducing before succumbing to inescapable and unpredictable disease-related mortality. Consistent with this logic are results suggesting that people with a history of greater vulnerability to illness more heavily discount the future, and report a lower ability to delay gratification^68^. Further, several studies suggest that adolescents with chronic illnesses initiate sexual behaviours earlier in their lives and are more likely to engage in more promiscuous and risky sexual behaviour^69–71^. Other work has found that adults diagnosed with a life-threatening chronic illness during childhood (e.g., cancer or epilepsy) employ faster reproductive strategies^72^, as do adults exposed to greater familial mortality during childhood^73^. Hill et al.^74^ similarly found that experimentally inducing perceived risk of future disease threat was associated with a desire for a greater number of novel sexual partners. However, almost no work has investigated the potential link between genetic markers of disease vulnerability and calibration of these life history-relevant traits. A heterozygote advantage perspective of MHC diversity implies that greater heterozygosity should be associated with slower life history strategies.

To date, only one study has examined the link between MHC and life history strategy. Murray et al.^14^ investigated the relationship between MHC heterozygosity and “fast” versus slow life history-related sexual strategies in a sample of 180 healthy women. Consistent with the predictions implied by a life history framework, they found that women with higher levels of MHC heterozygosity reported significantly less favourable attitudes towards short-term sexual behaviour, less actual past short-term sexual behaviour (assessed by one night stands, past-year partners, and lifetime partners), and a significantly later age at sexual debut. However, this study was limited in several respects. First, the dependent measures in the study were purely sexuality-based and thus assess only one specific aspect of life history strategy; assessing a fuller repertoire of life history indicators (such as impulsivity and future discounting) is required for a better understanding of this relationship. A second limitation pertains to the study’s small sample size; although often limited for pragmatic reasons, such underpowered sample sizes tend to be vulnerable to producing both Type 1 and 2 errors and also tend to lead to gross overestimates of “real” effect sizes in the published literature, especially in behavioural genetics research (e.g.,^75–77^). Third, the sample was comprised entirely of women, and only a minority of these women reported being heterosexual (one of the two data collection sites was a Pride event).

In the current study, we more comprehensively investigate the relationship between MHC heterozygosity and life history strategy by recruiting a larger sample (final *N* = 789) consisting of both men and women and assessing a fuller complement of life history indicators. We also tested whether MHC status was associated with reported health, dispositional disease-relevant affect (i.e. trait disgust), and self-perceived vulnerability to disease. Hypotheses, methods, and analyses were preregistered on aspredicted.org (aspredicted.org/blind.php?x=bi8tw6).

## Results

### Is MHC homozygosity associated with “faster” sexual strategies?

Zero-order correlations between the outcome variables of interest are shown in Table 1. In order to investigate the potential effect of homozygosity on life history-relevant sexual strategies, we conducted a multivariate analysis of variance entering homozygosity (1 = homozygous, 0 = heterozygous) as the predictor, and both the short-term mating attitudes composite and short-term sexual behaviours composite as outcome variables. Results from this analysis revealed no effect of MHC on the outcome variables simultaneously (Wilk’s Λ = 0.99, *F*(2,784) = 0.23, *p* = 0.80, partial *η*^2^ = 0.001. Results of the preregistered multiple analysis of covariance including the control variables of gender, ethnicity (both entered as fixed factors), and age (entered as a covariate) are shown in Table 2. As can be seen from the table, whereas each of the control variables significantly predicted variation in sexual strategies (*p*’s < 0.05), MHC did not (Wilk’s Λ = 0.99, *p* = 0.69, partial *η*^2^ = 0.001). Investigating at the outcome variables separately, neither SOI attitudes nor behaviours individually meaningfully differed in either analysis, *p*’s > 0.40 (see also raw mean comparisons in Table 3). No differences emerged when analysing men and women separately in either the multivariate test or when analysing the two SOI variables separately, or when excluding non-heterosexual participants (*p*’s > 0.20).

**Table 1:** Zero-order correlations between pertinent variables of interest.

Variable	1	2	3	4	5	6	7	8	9	10
1. Mini K	–									
2. SOI—attitudes	− 0.30***	–								
3. SOI—behaviour	− 0.20***	0.47***	–							
4. Delay of gratification	0.35***	− 0.16***	− 0.08*	–						
5. PVD—GA	0.16***	− 0.21***	− 0.14***	0.07*	–					
6. PVD—PI	0.01	0.01	0.01	− 0.11**	0.22***	–				
7. TDD—pathogen	0.19***	− 0.09*	0.00	− 0.04	0.36***	0.17***	–			
8. TDD—sexual	0.29***	− 0.49***	− 0.27	0.08*	0.27***	0.12**	0.47***	–		
9. TDD—moral	0.25***	− 0.20***	− 0.12**	0.19***	0.22***	0.05	0.36***	0.36***	–	
10. Child SES	0.11**	0.10**	0.08*	0.02	0.01	0.06	0.01	− 0.01	0.01	–
11. Child health	0.11**	− 0.01	− 0.03	0.15***	− 0.07*	− 0.46***	0.02	0.01	0.03	− 0.01

*p < .05; **p < .01; ***p < .001.

**Table 2:** Coefficients, estimated effect sizes, and *p*-values from MANCOVA results for each of the four variables simultaneously predicting SOI attititudes and SOI behaviours.

Predictor variable	Wilk’s Λ	Effect size (partial *η*^2^)	*p*
MHC heterozygosity	0.99	0.001	0.69
Ethnicity	0.94	0.029	< 0.001
Sex	0.96	0.041	< 0.001
Age	0.99	0.008	0.044

**Table 3:** Means (and standard deviations) of outcome variables for MHC-heterozygous and -homozygous participants, along with *p*-values of mean differences.

Variable	MHC-heterozygous	MHC-homozygous	*p* of difference (uncorrected)
Attitudes towards short-term mating	5.63 (2.27)	5.50 (2.28)	0.47
Short-term sexual behaviours (composite *z*-score)	− 0.03 (0.70)	− 0.04 (0.68)	0.84
Life history battery (Mini-K)	5.27 (0.66)	5.28 (0.62)	0.76
Delay of gratification	3.60 (0.54)	3.60 (0.54)	0.86
Sexual disgust	3.59 (1.26)	3.78 (1.33)	0.07
Pathogen disgust	4.50 (1.18)	4.52 (1.16)	0.83
Moral disgust	4.63 (1.15)	4.78 (0.99)	0.09
PVD germ aversion	3.86 (0.97)	3.80 (1.05)	0.47
PVD perceived infectibility	3.61 (1.32)	3.62 (1.29)	0.89
Childhood health (composite)	5.75 (1.47)	5.71 (1.44)	0.73
Current health	3.64 (0.79)	3.61 (0.82)	0.61

Some research suggests that faster sexual strategies necessitate a downregulation of disgust (e.g.,^78^). We thus tested whether MHC homozygous versus heterozygous participants reported lower dispositional disgust and lower perceived vulnerability to disease. These mean comparisons are shown in Table 3. As can be seen from the table, these groups did not meaningfully differ in pathogen disgust (*p* > 0.50). Although the (family-wise uncorrected) two-tailed *p*’s implied that these groups differed marginally-significantly on sexual and moral disgust, these differences are in the opposite direction implied by a life history framework (i.e. homozygous participants scored *higher* in disgust). Similarly, participants did not meaningfully differ in Germ Aversion or Perceived Infectability, *p*’s > 0.45. In women alone, none of these five variables differed by MHC group, *p*’s > 0.30. In men, MHC homozygous participants were marginally higher in moral disgust relative to MHC heterozygous participants (4.74 vs. 4.42; *t*(272) = 1.92, uncorrected *p* = 0.056). Again, however, this difference is in the opposite direction of that implied by a life history framework. No other differences approached significance (*p*’s > 0.12).

### Is MHC associated with other life history indicators?

At the root of the conceptual logic linking MHC diversity to life history strategies is the assumption that MHC status is an internal characteristic influencing vulnerability to novel pathogens and, ultimately, health. However, no measures of participant health (childhood or current) differed by MHC group (*p*’s > 0.50, see Table 3). This was also true when comparing MHC groups in men and women separately (all *p*’s > 0.14). Similarly, across the full sample both the general life history measure (Mini-K) and Delay of Gratification did not differ by MHC status (*p*’s > 0.50). This was similarly the case when analysing both men and women separately (all *p*’s > 0.17).

### Is MHC associated with Big Five personality traits?

Consistent with previous research investigating the Big Five personality correlates of life history strategy (e.g.,^79,80^), participants on the “slower” end of the life history spectrum (measured by the Mini-K) were higher in conscientiousness (*r*(789) = 0.41, *p* < 0.001), agreeableness (*r* = 0.38, *p* < 0.001), and extraversion (*r* = 0.32, *p* < 0.001). Slower life history scores were correlated only minimally with neuroticism (*r* = − 0.07, *p* = 0.049) and openness (*r* = 0.06, *p* = 0.10). Similar to the results above, however, MHC-homozygous and -heterozygous participants did not differ on any of the Big Five traits, *t*’s < 1.30, *p*’s > 0.19.

Analysing women and men separately, MHC homozygous and heterozygous women did not differ on any of the Big Five traits, *p*’s > 0.30. MHC homozygous men did report significantly higher levels of neuroticism [3.08 vs. 2.71, *t*(272) = 3.13, *p* = 0.002], and marginally higher levels of extraversion [3.39 vs. 3.18, *t*(272) = 1.81, *p* = 0.071]. This neuroticism difference is the one significant result consistent with differences implied by the MHC heterozygosity/slower life history framework.

### Additional analyses

In light of the null results reported above, we performed several additional sets of exploratory analyses designed to further test for evidence of any effect of MHC diversity within the current sample. The first set of analyses assessed whether any group differences emerged between individuals who are heterozygous or homozygous at each of the three specific loci tested here. We performed independent samples *t*-tests to test for any evidence of mean differences between heterozygous and homozygous participants for each of the eleven outcome variables reported in Table 3, as well as for each Big Five personality factor. Full results of these analyses are reported in Supplementary Tables S1–S3. All reported *p*’s are uncorrected for multiple tests.

Ninety-three participants were homozygous (696 heterozygous) at the HLA-A locus. Independent *t*-tests on each of the eleven DV’s revealed no significant differences between hetero-and homozygous participants, absolute *t*’s < 1.3, *p*’s > 0.20 (see Table S1). Despite the lower power (from unbalanced groups) of these tests, no evidence for any pattern emerged: examining the raw means revealed that four of the nonsignificant differences were in the direction implied by life history theory, six in the opposite direction, and one no difference (to the third decimal place). Similarly, no differences in Big Five personality scores emerged, *p*’s > 0.17.

Fifty-eight participants were homozygous (731 heterozygous) at the HLA-B locus. Independent t-tests on each of the eleven DV’s revealed no significant differences between hetero-and homozygous participants, absolute *t*’s < 1.2, *p*’s > 0.23 (see Table S2). Despite the lower power (from unbalanced groups) of these tests no evidence for any pattern emerged: examining the raw means revealed that three of the nonsignificant differences were in the direction implied by life history theory, and eight in the opposite direction. Two notable differences (both in the direction implied by the theoretical framework) did emerge in the analyses of personality traits, with homozygous participants being lower in conscientiousness (3.39 vs. 3.58, *t* = − 2.02, *p* = 0.044), and higher in neuroticism (3.27 vs. 3.02, *t* = 2.32, *p* = 0.020). However, the significance of these differences would not survive even the most liberal correction for multiple tests (and, the preregistration set the significance threshold for even the original more limited set of exploratory analyses at *p* = 0.01).

Ninety-one participants were homozygous (698 heterozygous) at the HLA-DRB1 locus. Independent t-tests on each of the eleven DV’s revealed no significant differences between hetero-and homozygous participants, absolute *t*’s < 1.3, *p*’s > 0.20 (see Table S3). No evidence for any consistent pattern emerged among the nonsignificant mean differences: eight of the nonsignificant differences were in the direction implied by life history theory, and three in the opposite direction. Similarly, no differences in Big Five personality scores emerged, *p*’s > 0.13.

Finally, given that dichotomizing MHC homozygosity removes potentially meaningful variation from the predictor variable, it could be argued that correlational tests offer a better analytical strategy to test the relationship between MHC homozygosity and the outcome variables of interest (an analytical strategy employed by Lie et al.^3^, albeit with a greater number of loci). We thus ran correlational tests (Spearman’s *ρ*) between degree of MHC homozygosity (i.e., homozygous at 0, 1, 2, or 3 loci) and the eleven dependent measures. Results of these correlational analyses revealed no evidence for significant relationships in any direction (absolute *ρ*’s < 0.07, *p*’s > 0.07; largest relationship with sexual disgust, *ρ* = 0.062, *p* = 0.077; all other *p*’s > 0.21). Parallel correlational analyses testing women and men separately similarly revealed no evidence for any meaningful relationships (in women, *ρ*’s < 0.08, *p*’s > 0.07; in men, *ρ*’s < 0.10, *p*’s > 0.11). Similar to the dichotomized analyses, correlational analyses with the Big Five personality traits revealed a significant positive relationship between MHC homozygosity and neuroticism in men (*ρ* = 0.175, *p* = 0.004), but no other significant relationships in either women (*p*’s > 0.25) nor in women and men analysed together (*p*’s > 0.17).

## Discussion

The results can be summarized as follows: higher MHC heterozygosity was not associated with any indicators of life history strategy, including sexual strategy, impulsivity, and the Life History battery itself. Similarly, MHC was not associated with current or childhood health, dispositional worry about disease, or disgust propensity. Further, it was largely unrelated to oblique correlates of life history such as the Big Five personality traits. This was true for both men and women.

Several limitations of the current study—and how they may have contributed to the non-effects reported here—deserve note. First, although the current sample size was larger than that from previous MHC-related work, it was not in the realm of molecular genetics work that is now able to employ samples that number tens of thousands of participants. At the very least, however, the current results suggest that previous findings linking MHC homozygosity to sexual behaviour represent overestimates of such effects, should they in fact be real. Indeed, as behavioural genetics research has proliferated over the past decade, one of the meta-conclusions that has emerged is that in most cases, the effects of one allelic variant on complex traits are likely to be very small (e.g.,^76,81,82^). Although the current study did not investigate specific polymorphic variation per se, the same may be true when investigating the MHC: “normal” variation in levels of heterozygosity may simply have small or negligible effects on life history-relevant cognition and behaviour.

Second, the current sample consisted of young, healthy, and wealthy participants enrolled in a private institution in the southern United States. This poses an issue for studying putative genetic effects on both health and life history; most likely, these individuals developed in relatively safe and controllable environments, and thus have simply not received the environmental inputs (stressors, deadly pathogenic threats, etc.) that would make the immune-related consequences of MHC heterozygosity apparent. Although previous research suggests that when investigating the major histocompatibility complex the ethnicity of the sample should be restricted to reduce confounds (see^48^), the limited range of ethnic backgrounds of the participants may have also been a factor. Previous research—which reported mixed results for MHC heterozygosity—found that MHC-dissimilarity mating was only found in couples for which both members were Asian^38^. Another potential reason why these results differ specifically from previous preliminary findings pertains to sexual orientation: whereas the current sample overwhelmingly reported being heterosexual, only a minority of Murray and colleagues’^14^ sample (about 40%) reported being heterosexual. Future research may thus discover more nuanced links between disease vulnerability, sexual strategies, and sexual orientation itself.

A third limitation in this study pertains to the actual genotyping of the major histocompatibility complex. Consistent with the most closely-related related studies (e.g.,^14,48^), the current study involved studying diversity at the three classic (and putatively most polymorphic) MHC loci—HLA-A, HLA-B, and HLA-DRB1. Although these are the loci that have been investigated most frequently in influential studies investigating the implications of the MHC for human cognition and behaviour (e.g.,^37,39–41,48^), they fail to fully capture diversity across the entire MHC. Other studies investigating the implications of MHC for mate choice have found associations at the higher-resolution, single-nucleotide polymorphism (SNP) level of analysis^8^. Future research may thus find a more nuanced relationship between MHC diversity and life history characteristics at more fine-grained levels of analysis.

Finally, in light of the results reported here it is necessary to consider that some piece of the underlying theoretical framework linking MHC to life history is simply incorrect, and that earlier positive results attesting to this relationship were merely spurious. It is possible that MHC heterozygosity—no matter how it is measured—is inconsequential for life history calibration. More fundamentally, it is worth considering whether the levels of MHC heterozygosity in the current sample represent local optima (e.g.,^22,23^), and thus slight variations have little consequence for domain-general disease morbidity and mortality, or whether specific alleles rather than diversity per se carry adaptive value due to pathogen-specific selection (e.g.,^32^). Finally, despite its recent popularization in the behavioural sciences, it is also possible that a life history framework has its major utility in studying inter-*species* variation (as it was originally intended) but has limited utility for studying inter-*individual* behavioural variation, or ephemeral variation at different time points e.g., (see^83^).

It is also worth noting that intuitive logic may arrive at the opposite prediction than that presented here—that higher vulnerability to disease may lead to a *less* promiscuous sexual strategy as a behaviourally-protective measure against human-transmitted infections, delayed reproductive timing as a result of increased metabolic allocation to somatic maintenance, and greater affective aversions towards pathogenic vectors (e.g., higher disgust). Indeed, at the psychological level, some research suggests that trait-like aversion to germs and disease vectors is associated with less promiscuous sexual attitudes and behaviours^84–86^. However, there appears to be little to no relationship between actual disease vulnerability and dispositional disgust (e.g.,^74,87,88^). Both these pragmatic and conceptual limitations and considerations should inform future work.

Despite these limitations, the current work represents the largest study to date directly designed to investigate the potential relationship between diversity at the MHC and life history strategy. The current results suggest that—at least for healthy individuals who grew up in relatively stable ecologies—MHC may not meaningfully influence life history trajectories or the indicators thereof. Future research, employing more sophisticated genetic analyses and recruiting participants from harsher and more uncertain ecologies, is needed to further investigate the relationships between MHC diversity, disease vulnerability, and life history calibration.

## Methods

Data from the study are available at osf.io/tp6dr/?view_only=39b2e1ffa28449578b431162e3180b43. The study was approved by Tulane University’s Biomedical Institutional Review Board (IRB# 16-908071). All methods were carried out in compliance with institutional and federal regulations. Informed consent was obtained from all participants before beginning the study.

### Participants

Participants were 789 undergraduates (65.3% women; 87.9% heterosexual; mean age = 18.9 years, SD = 0.95) from a southern university in the United States, who participated in exchange for partial course credit. Data collection took place over the course of two calendar years. Original sample size was predetermined as 800 based upon project-specific funding; this sample size provided statistical power of 0.90 to detect an effect size of *d* = 0.25 between MHC heterozygous and homozygous participants if an estimated 30% of the sample was classified as homozygous (see below). Three participants reported being under the age of 18, five participants’ saliva samples could not be sequenced, and three participants did not complete the survey, leaving a useable final sample of 789 participants. Using an open-response ethnicity question, 73.4% reported being White/European, 10.6% Asian, 4.3% Black/African American, 4.1% Latinx, 6.5% mixed ethnicity, and 1.1% other ethnicities.

### Procedure

Upon arrival to the lab, participants provided written consent and were assigned a unique participant ID (in order to link questionnaire and biological specimen data). Participants were seated in a private room to complete the full set of questionnaires, described below. Upon completion of the questionnaires, participants provided their saliva sample for genotyping. Having participants first complete the questionnaires ensured that participants refrained from eating and drinking for at least 15 min prior to providing their saliva sample. Participants provided saliva via passive drool using an industry-standard Oragene® saliva collection kit (DNA Genotek Inc., Ottawa, ON, Canada). If a participant complained of an inability to produce sufficient saliva, they were given the option of using a small amount (just a dab on their moistened fingertip) of artificial sweetener e.g., (Equal, Sweet ‘n Low) to stimulate saliva production. Following the saliva sample, participants were debriefed and were free to leave (see^14^ for original description of the procedures and measures).

### Measures

Participants completed several life-history-related questionnaires, described below.

#### Sexual strategy

We assessed participants’ attitudes toward short-term mating using the three attitudinal items from Simpson and Gangestad’s^89^ Sociosexual Orientation Inventory: “Sex without love is OK,” “I can imagine myself being comfortable with and enjoying ‘casual’ sex with different partners,” and “I would have to be closely attached to someone (both emotionally and psychologically) before I could feel comfortable and fully enjoy having sex with him or her” (reverse scored). Participants indicated their agreement with these items on a 9-point Likert scale. Participants’ responses to the three items were highly intercorrelated (*r*’s > 0.50, α = 0.82) and were combined to create a single short-term mating attitudes score, with higher scores indicating more favourable attitudes toward short-term mating. Second, we created a short-term mating behaviour score, with higher scores indicating a more promiscuous/short-term sexual history. This consisted of the composite of participants’ self-reported lifetime number of sex partners, number of sex partners in the past year, and number of one-time sex partners. These values were highly intercorrelated, *r*’s > 0.73, α = 0.91). As dictated by the preregistered analysis plan, in order to reduce positive skew for these free-response answers any *z*-scores above 3 for any of these individual items were winsorized to the next highest score (1.4% or 34 total responses). The data collection plan also dictated asking participants when in their lives they first engaged in sexual activity. However, due to a survey software error no participants completed this question.

#### Life history strategy

Life history strategy (the “K-Factor”) was assessed using the Mini-K, which aims to capture the comorbid cluster of familial, sexual, and social attitudes and behaviours associated with slower life history strategies^90^. Participants indicated their agreement to 20 items (e.g., “I often make plans in advance”; “I would rather have one than several sexual relationships at a time”) on a scale of − 3 (Disagree Strongly) to + 3 (Agree Strongly; α = 0.75). A higher score on the mean scored composite indicated that a participant endorsed more attitudes and behaviours consistent with following a slow life history strategy.

#### Delay of gratification

Participants completed the short-form Delay of Gratification Inventory^91^, comprised of 10 items (e.g., “I would have a hard time sticking with a special, healthy diet”) rated on a scale of 1 (Strongly Disagree) to 5 (Strongly Agree). Reliability was modest, α = 0.65. A higher score on this composite would indicate planning behaviour consistent with a slower life history strategy.

#### Disgust

Participants completed the Three Domain Disgust scale^86^, which assesses reactions to pathogen-related (e.g., “Accidentally touching a person's bloody cut”), sex-related (e.g., “Hearing two strangers having sex”), and moral-related (e.g., “Intentionally lying during a business transaction”) disgust reactions on a scale of 0 (not at all disgusting) to 6 (extremely disgusting). Reliabilities of each subscale were good, α’s > 0.80.

#### Dispositional worry about disease threat

Participants completed the 15-item Perceived Vulnerability to Disease Scale, which is designed to assess participants’ dispositional concern about disease threats^84^. The questionnaire is comprised of two subscales. The 8-item Germ Aversion (PVD-GA; α = 0.74) subscale measures individuals’ discomfort in situations that imply a probability of disease transmission (e.g., “I don’t like to write with a pencil someone else has obviously chewed on”). The 7-item Perceived Infectability (PVD-PI; α = 0.92) subscale measures the individuals’ degree to which they believe they are vulnerable to contracting infectious diseases (e.g., “I am more likely than the people around me to catch an infectious disease”). Participants rated their agreement on a 7-point scale ranging from ‘Strongly Disagree’ to ‘Strongly Agree.’ Consistent with previous research (e.g.,^84^) these two subscales were only modestly correlated, *r* = 0.22, *p* < 0.001.

#### Personality

Personality was assessed using the 44-item Big Five Inventory^92^, which assesses extraversion, agreeableness, conscientiousness, openness to experience, and neuroticism. Participants rated each item on a five-point scale, from ‘Disagree Strongly’ to ‘Agree Strongly’. Reliabilities for each factor were good, α’s > 0.75.

#### Health

Participants completed several measures assessing various aspects of health. These included global single-item estimates of current health (response options: poor, fair good, very good, excellent), along with three items assessing childhood health (e.g., “When I was growing up, I missed a lot of school due to illness”; 1 = Strongly Agree, 7 = Strongly Disagree; α = 0.93; taken from 74).

Participants also filled completed a set of demographic questions (e.g., childhood SES). Correlations between the outcome variables of interest are shown in Table One. As can be seen from the table, the interrelationships between the variables are consistent with previous work (e.g.,^79,80,90,93,94^).

### Genotyping

In compliance with manufacturer protocols, immediately after collection the saliva samples were mixed with each kits’ stabilizing solution and stored at room temperature. Samples were shipped for extraction and genotyping twice over the course of data collection in order to decrease chances of DNA degradation and to ensure appropriate DNA yield for earlier-collected kits. Samples were shipped to Histogenetics® (Ossining, NY) for extraction and for Next Generation-based HLA typing at three loci: HLA-A, HLA-B and HLA-DRB1 (using Illumina HiSeq/MiSeq platform; quality score Q30, base call accuracy 99.9%). These are the three “classic” MHC genes most commonly typed in similar investigations (e.g.,^14,48^; for more details on HLA sequencing and analysis, see^95^).

Typing results revealed that 587 participants (74.4%) were heterozygous at all three loci. Of the 202 participants that were homozygous at any locus, 168 were homozygous at just one locus, 26 were homozygous at two loci, and 6 were homozygous at all three loci. In total, 88.6% of typed loci were heterozygous, similar to previous work, [e.g., 87.5% from^3^; 88.7% in^14^]. Consistent with previous related research (e.g.,^14,48^), we characterized participants as “homozygous” if they were homozygous at one or more loci.

### Statistical methods

All analyses were performed using SPSS (version 26). Following the analytical strategy used by Murray et al.^14^, the preliminary preregistered analyses was a multivariate analysis of variance (MANOVA) investigating the simultaneous predictive effects of MHC heterozygosity (fixed factor; dichotomized as homozygous or heterozygous) on both short-term mating attitudes and previous short-term mating behaviours. The preregistered follow-up analysis of covariance (ANCOVA) entered MHC heterozygosity as a fixed factor along with biological sex (male, female) as a fixed factor, ethnicity as a fixed factor (coded as: White/European, Asian, Black/African American, Native American/Alaskan, Mixed ethnicity, Other), and age (in years) entered as a covariate in predicting the same SOI attitude and behaviour outcome variables. As we had no a priori predictions about interactions between MHC and these other predictors, the model reported here was constrained to only main effects. Subsequent follow-up analyses of the full factorial model which included all interaction terms between the fixed factors revealed no evidence for a three-way interaction (Wilk’s Λ = 0.995, *p* = 0.89), nor any two-way interactions (*p*’s > 0.70). Finally, the preliminary data analysis plan for more exploratory outcome variables simply involved mean comparisons between homozygous and heterozygous participants using independent samples *t*-tests, with the preregistered significance threshold set at *p* = 0.01.

## Supplementary information

Supplementary information.

## Data Availability

Data for this study are available at osf.io/tp6dr/?view_only=39b2e1ffa28449578b431162e3180b43.

## References

[1] Burger D, Dolivo G, Marti E, Sieme H, Wedekind C (2015). Female major histocompatibility complex type affects male testosterone levels and sperm number in the horse (*Equus caballus*). Proc. R. Soc. B.

[2] Burger D (2017). Major histocompatibility complex-linked social signalling affects female fertility. Proc. R. Soc. B.

[3] Lie HC, Simmons LW, Rhodes G (2010). Genetic dissimilarity, genetic diversity, and mate preferences in humans. Evol. Hum. Behav..

[4] Probst F, Fischbacher U, Lobmaier JS, Wirthmüller U, Knoch D (2017). Men's preferences for women's body odours are not associated with human leucocyte antigen. Proc. R. Soc. B.

[5] Ruff JS, Nelson AC, Kubinak JL, Potts WK, López-Larrea C (2012). MHC signaling during social communication. Self and Nonself.

[6] Sorokowska A (2018). Human Leukocyte Antigen similarity decreases partners' and strangers' body odor attractiveness for women not using hormonal contraception. Horm. Behav..

[7] Winternitz J, Abbate JL, Huchard E, Havlíček J, Garamszegi LZ (2017). Patterns of MHC-dependent mate selection in humans and nonhuman primates: a meta-analysis. Mol. Ecol..

[8] Wu K (2018). More than skin deep: Major histocompatibility complex (MHC)-based attraction among Asian American speed-daters. Evol. Hum. Behav..

[9] Abbas AK, Lichtman AH, Pillai S (2014). Basic Immunology: Functions and Disorders of the Immune System.

[10] Davies DM (2013). The Compatibility Gene.

[11] Bhutta MF (2007). Sex and the nose: human pheromonal responses. J. R. Soc. Med..

[12] Oliver MK, Telfer S, Piertney SB (2008). Major histocompatibility complex (MHC) heterozygote superiority to natural multi-parasite infections in the water vole (*Arvicola terrestris*). Proc. R. Soc. B.

[13] Penn DJ, Damjanovich K, Potts WK (2002). MHC heterozygosity confers a selective advantage against multiple-strain infections. Proc. Natl. Acad. Sci. USA.

[14] Murray DR, Gildersleeve KA, Fales MR, Haselton MG (2017). MHC homozygosity is associated with fast sexual strategies in women. Adapt. Hum Behav. Physiol..

[15] Murphy K, Weaver C (2016). Janeway's Immunobiology.

[16] Cooke GS, Hill AVS (2001). Genetics of susceptibitlity to human infectious disease. Nat. Rev. Genet..

[17] McLelland EE, Penn DJ, Potts WK (2003). Major histocompatibility complex heterozygote superiority during coinfection. Infect. Immun..

[18] Carrington M, O’Brien SJ (2003). The influence of HLA genotype on AIDS. Annu. Rev. Med..

[19] Osborne AJ, Pearson J, Negro SS, Chilvers BL, Kennedy MA, Gemmell NJ (2015). Heterozygote advantage at MHC *DRB* may influence response to infectious disease epizootics. Mol. Ecol..

[20] Kurtz J (2004). Major histocompatibility complex diversity influences parasite resistance and innate immunity in sticklebacks. Proc. R. Soc. B.

[21] Doherty PC, Zinkernagel RM (1975). A biological role for the major histocompatibility antigens. Lancet.

[22] Nowak MA, Tarczy-Hornoch K, Austyn JM (1992). The optimal number of major histocompatibility complex molecules in an individual. Proc. Natl. Acad. Sci. USA.

[23] Woelfing B, Traulsen A, Milinski M, Boehm T (2009). Does intra-individual major histocompatibility complex diversity keep a golden mean?. Philos. Trans. R. Soc. Lond. B Biol. Sci..

[24] Wegner KM, Kalbe M, Kurtz J, Reusch TB, Milinski M (2003). Parasite selection for immunogenetic optimality. Science.

[25] Bonneaud C, Mazuc J, Chastel O, Westerdahl H, Sorci G (2004). Terminal investment induced by immune challenge and fitness traits associated with major histocompatibility complex in the house sparrow. Evolution.

[26] Kalbe M (2009). Lifetime reproductive success is maximized with optimal major histocompatibility complex diversity. Proc. R. Soc. B.

[27] Madsen T, Ujvari B (2006). MHC class I variation associates with parasite resistance and longevity in tropical pythons. J. Evol. Biol..

[28] Langefors Å, Lohm J, Grahn M, Andersen Ø, Schantz TV (2001). Association between major histocompatibility complex class IIB alleles and resistance to *Aeromonas salmonicida* in Atlantic salmon. Proc. R. Soc. B.

[29] Paterson S, Wilson K, Pemberton JM (1998). Major histocompatibility complex variation associated with juvenile survival and parasite resistance in a large unmanaged ungulate population (*Ovis aries* L.). Proc. Natl. Acad. Sci. USA.

[30] Wedekind C, Walker M, Little TJ (2005). The course of malaria in mice: major histocompatibility complex (MHC) effects, but no general MHC heterozygote advantage in single-strain infections. Genetics.

[31] Lohm J (2002). Experimental evidence for major histocompatibility complex–allele–specific resistance to a bacterial infection. Proc. R. Soc. B.

[32] Consuegra S, Garcia de Leaniz C (2008). MHC-mediated mate choice increases parasite resistance in salmon. Proc. R. Soc. B.

[33] O'Dwyer T, Nevitt G (2009). Individual odor recognition in procellariiform chicks. Ann. NY Acad. Sci..

[34] Penn D, Potts W (1998). How do major histocompatibility complex genes influence odor and mating preferences?. Adv. Immunol..

[35] Schwensow N, Eberle M, Sommer S (2007). Compatibility counts: MHC-associated mate choice in a wild promiscuous primate. Proc. R. Soc. B.

[36] Yamazaki K, Beauchamp GK, Curran M, Bard J, Boyse EA (2000). Parent–progeny recognition as a function of MHC odortype identity. Proc. Natl. Acad. Sci. USA.

[37] Garver-Apgar CE, Gangestad SW, Thornhill R, Miller RD, Olp JJ (2006). Major histocompatibility complex alleles, sexual responsivity, and unfaithfulness in romantic couples. Psychol. Sci..

[38] Saphire-Bernstein S (2017). Genetic compatibility in long-term intimate relationships: partner similarity at major histocompatibility complex (MHC) genes may reduce in-pair attraction. Evol. Hum. Behav..

[39] Thornhill R (2003). Major histocompatibility complex genes, symmetry, and body scent attractiveness in men and women. Behav. Ecol..

[40] Wedekind C, Seebeck T, Bettens F, Paepke AJ (1995). MHC-dependent mate preferences in humans. Proc. R. Soc. B.

[41] Wedekind C, Füri S (1997). Body odour preferences in men and women: do they aim for specific MHC combinations or simply heterozygosity?. Proc. R. Soc. B.

[42] Edwards SV, Hedrick PW (1998). Evolution and ecology of MHC molecules: from genomics to sexual selection. Trends Ecol. Evol..

[43] Chaix R, Cao C, Donnelly P (2008). Is mate choice in humans MHC-dependent?. PLoS Genet..

[44] Laurent R, Chaix R (2012). MHC-dependent mate choice in humans: Why genomic patterns from the HapMap European American dataset support the hypothesis. BioEssays.

[45] Ober C (1997). HLA and mate choice in humans. Am. J. Med. Genet..

[46] Derti A, Cenik C, Kraft P, Roth FP (2010). Absence of evidence for MHC–dependent mate selection within HapMap populations. PLoS Genet..

[47] Hedrick PW, Black FL (1997). HLA and mate selection: no evidence in South Amerindians. Am. J. Hum. Genet..

[48] Roberts SC (2005). MHC-assortative facial preferences in humans. Biol. Lett..

[49] Coetzee V (2007). Common HLA alleles associated with health, but not with facial attractiveness. PLoS ONE.

[50] Havlicek J, Roberts SC (2009). MHC-correlated mate choice in humans: a review. Psychoneuroendocrinology.

[51] Belsky J, Steinberg L, Draper P (1991). Childhood experience, interpersonal development, and reproductive strategy: an evolutionary theory of socialization. Child Dev..

[52] Chisholm JS (1999). Death, Hope and Sex.

[53] Del Giudice M (2009). Sex, attachment, and the development of reproductive strategies. Behav. Brain. Sci..

[54] Ellis BJ, Figueredo AJ, Brumbach BH, Schlomer GL (2009). Fundamental dimensions of environmental risk. Hum. Nat..

[55] Griskevicius V (2013). Economic recessions, childhood environments, and the contingent expression of fast and slow life history strategies. Psychol. Sci..

[56] Kaplan HS, Gangestad SW, Buss DM (2005). Life history theory and evolutionary psychology. The Handbook of Evolutionary Psychology.

[57] MacDonald K (1997). Life history theory and human reproductive behavior. Hum. Nat..

[58] Belsky J (2012). The development of human reproductive strategies: progress and prospects. Curr. Dir. Psychol. Sci..

[59] Belsky J, Steinberg L, Houts RM, Halpern-Fisher BL (2010). The development of reproductive strategy in females: early maternal harshness → earlier menarche → increased sexual risk taking. Dev. Psychol..

[60] Chisholm JS (1999). Attachment and time preference. Hum. Nat..

[61] Griskevicius V (2013). When the economy falters, do people spend or save? Responses to resource scarcity depend on childhood environments. Psychol. Sci..

[62] Nettle D (2011). Behaviour of parents and children in two contrasting urban neighbourhoods: an observational study. J. Ethol..

[63] Nettle D, Cockerill M (2010). Development of social variation in reproductive schedules: a study from an English urban area. PLoS ONE.

[64] Wilson M, Daly M (1997). Life expectancy, economic inequality, homicide, and reproductive timing in Chicago neighbourhoods. BMJ Brit. Med. J..

[65] Quinlan RJ (2006). Human parental effort and environmental risk. Proc. R. Soc. B.

[66] Pelham B (2019). Life history and the cultural evolution of parenting: Pathogens, mortality, and birth across the globe. Evol. Behav. Sci..

[67] Low BS (1990). Marriage systems and pathogen stress in human societies. Am. Zool..

[68] Hill SE, Boehm GW, Prokosch ML (2016). Vulnerability to disease as a predictor of faster life history strategies. Adapt. Hum. Behav. Physiol..

[69] Choquet M, Du Pasquier D, Fediaevsky L, Manfredi R (1997). Sexual behavior among adolescents reporting chronic conditions: a French national survey. J. Adolesc. Health.

[70] Suris JC, Michaud PA, Akre C, Sawyer SM (2008). Health risk behaviors in adolescents with chronic conditions. Pediatrics.

[71] Suris JC, Parera N (2005). Sex, drugs and chronic illness: health behaviours among chronically ill youth. Eur. J. Public Health.

[72] Waynforth D (2012). Life-history theory, chronic childhood illness and the timing of first reproduction in a British birth cohort. Proc. R. Soc. B.

[73] Störmer C, Lummaa V (2014). Increased mortality exposure within the family rather than individual mortality experiences triggers faster life-history strategies in historic human populations. PLoS ONE.

[74] Hill SE, Prokosch ML, DelPriore DJ (2015). The impact of perceived disease threat on women’s desire for novel dating and sexual partners: Is variety the best medicine?. J. Pers. Soc. Psychol..

[75] Button KS (2013). Power failure: why small sample size undermines the reliability of neuroscience. Nat. Rev. Neurosci..

[76] Dick DM (2015). Candidate gene–environment interaction research. Perspect. Psychol. Sci..

[77] Simmons JP, Nelson LD, Simonsohn U (2011). False-positive psychology: undisclosed flexibility in data collection and analysis allows presenting anything as significant. Psychol. Sci..

[78] Al-Shawaf L, Lewis DM, Buss DM (2015). Disgust and mating strategy. Evol. Hum. Behav..

[79] Figueredo AJ, Vásquez G, Brumbach BH, Schneider SMR (2007). The K-factor, covitality, and personality. Hum. Nat..

[80] Manson JH (2017). Are extraversion and openness indicators of a slow life history strategy?. Evol. Hum. Behav..

[81] Kendler KS (2013). What psychiatric genetics has taught us about the nature of psychiatric illness and what is left to learn. Mol. Psychiatry.

[82] Sullivan PF, Daly MJ, O'Donovan M (2012). Genetic architectures of psychiatric disorders: the emerging picture and its implications. Nat. Rev. Genet..

[83] Zietsch BP, Sidari MJ (2019). A critique of life history approaches to human trait covariation. Evol. Hum. Behav..

[84] Duncan LA, Schaller M, Park JH (2009). Perceived vulnerability to disease: development and validation of a 15-item self-report instrument. Pers. Ind. Differ..

[85] Murray DR, Jones DN, Schaller M (2013). Perceived threat of infectious disease and its implications for sexual attitudes. Pers. Ind. Differ..

[86] Tybur JM, Lieberman D, Griskevicius V (2009). Microbes, mating, and morality: individual differences in three functional domains of disgust. J. Pers. Soc. Psychol..

[87] De Barra M, Islam MS, Curtis V (2014). Disgust sensitivity is not associated with health in a rural Bangladeshi sample. PLoS ONE.

[88] Tybur JM (2016). Parasite stress and pathogen avoidance relate to distinct dimensions of political ideology across 30 nations. Proc. R. Soc. B.

[89] Simpson JA, Gangestad SW (1991). Individual differences in sociosexuality: evidence for convergent and discriminant validity. J. Pers. Soc. Psychol..

[90] Figueredo AJ (2005). The K-factor: Individual differences in life history strategy. Pers. Ind. Differ..

[91] Hoerger M, Quirk SW, Weed NC (2011). Development and validation of the delaying gratification inventory. Psychol. Assess..

[92] John OP, Srivastava S, Pervin L, John O (1999). The Big Five trait taxonomy: history, measurement, and theoretical perspectives. Handbook of Personality: Theory and Research.

[93] Manson JH (2015). Life history strategy and the HEXACO personality dimensions. Evol. Psychol..

[94] Strouts PH, Brase G, Dillon HM (2017). Personality and evolutionary strategies: The relationships between HEXACO traits, mate value, life history strategy, and sociosexuality. Pers. Ind. Differ..

[95] Shaw BE, Treleaven J, Barrett AJ (2009). Human leukocyte antigen matching, compatibility testing and donor selection. Hematopoietic Stem Cell Transplantation in Clinical Practice.

