# Supplementary material for: No evidence for a relationship between MHC heterozygosity and life history strategy in a sample of North American undergraduates
Source: Sci Rep. 2020 Jun 23;10:10140. doi: 10.1038/s41598-020-67406-7 (PMC7311407; doi:10.1038/s41598-020-67406-7)
Supplement: Supplementary file 1 — Supplementary information. [file 41598_2020_67406_MOESM1_ESM.pdf]

Supplementary Materials: No Evidence for a Relationship between MHC Heterozygosity and Life History Strategy in a Sample of North American Undergraduates

Damian R. Murray[1],

James B. Moran[1],

Marjorie L. Prokosch[1],

&

Nicholas Kerry[1]

[1] Department of Psychology, Tulane University, New Orleans, LA 70118

Table S1.

Means (and standard deviations) of outcome variables for participants heterozygous (n = 696) and homozygous (n = 93) at the HLA-A locus, along with p-values of mean differences.

Variable	HLA-A Heterozygous	HLA-A Homozygous	*p* of difference (uncorrected)
Attitudes towards Short-Term Mating	5.62 (2.27)	5.43 (2.29)	.46
Short-term Sexual Behaviours (composite *z*-score)	-0.03 (0.71)	-0.11 (0.60)	.27
Life History Battery (Mini-K)	5.27 (0.65)	5.25 (0.63)	.78
Delay of Gratification	3.60 (0.54)	3.60 (0.55)	.99
Sexual Disgust	3.62 (1.26)	3.77 (1.42)	.29
Pathogen Disgust	4.51 (1.18)	4.49 (1.17)	.93
Moral Disgust	4.65 (1.12)	4.80 (1.02)	.20
PVD Germ Aversion	3.85 (0.99)	3.84 (1.01)	.96
PVD Perceived Infectibility	3.62 (1.33)	3.55 (1.17)	.61
Childhood Health (composite)	5.73 (1.48)	5.84 (1.35)	.50
Current Health	3.64 (0.80)	3.61 (0.81)	.79

Table S2.

Means (and standard deviations) of outcome variables for participants heterozygous (n = 731) and homozygous (n = 58) at the HLA-B locus, along with p-values of mean differences.

Variable	HLA-B Heterozygous	HLA-B Homozygous	*p* of difference (uncorrected)
Attitudes towards Short-Term Mating	5.62 (2.28)	5.34 (2.22)	.37
Short-term Sexual Behaviours (composite *z*-score)	-0.03 (0.69)	-0.06 (0.80)	.78
Life History Battery (Mini-K)	5.27 (0.65)	5.22 (0.69)	.57
Delay of Gratification	3.60 (0.54)	3.55 (0.61)	.47
Sexual Disgust	3.63 (1.28)	3.77 (1.29)	.44
Pathogen Disgust	4.50 (1.17)	4.61 (1.20)	.49
Moral Disgust	4.66 (1.12)	4.78 (1.03)	.42
PVD Germ Aversion	3.86 (0.97)	3.70 (1.18)	.23
PVD Perceived Infectibility	3.61 (1.32)	3.60 (1.20)	.93
Childhood Health (composite)	5.73 (1.48)	5.89 (1.17)	.43
Current Health	3.64 (0.80)	3.64 (0.81)	.97

Table S3.

Means (and standard deviations) of outcome variables for participants heterozygous (n = 698) and homozygous (n = 91) at the HLA-DRB1 locus, along with p-values of mean differences.

Variable	HLA-DRB1 Heterozygous	HLA-DRB1 Homozygous	*p* of difference (uncorrected)
Attitudes towards Short-Term Mating	5.58 (2.28)	5.68 (2.28)	.71
Short-term Sexual Behaviours (composite *z*-score)	-0.05 (0.69)	0.05 (0.73)	.21
Life History Battery (Mini-K)	5.27 (0.66)	5.30 (0.56)	.59
Delay of Gratification	3.59 (0.54)	3.63 (0.54)	.51
Sexual Disgust	3.64 (1.29)	3.67 (1.26)	.83
Pathogen Disgust	4.51 (1.18)	4.45 (1.15)	.62
Moral Disgust	4.67 (1.13)	4.66 (0.98)	.97
PVD Germ Aversion	3.85 (0.99)	3.82 (1.00)	.80
PVD Perceived Infectibility	3.59 (1.29)	3.76 (1.45)	.26
Childhood Health (composite)	5.76 (1.44)	5.56 (1.64)	.22
Current Health	3.64 (0.80)	3.57 (0.83)	.43
